# Supplementary material for: Survival of sentinel node biopsy versus observation in intermediate-thickness melanoma: A Dutch population-based study
Source: PLoS One. 2021 May 25;16(5):e0252021. doi: 10.1371/journal.pone.0252021 (PMC8148374; doi:10.1371/journal.pone.0252021)
Supplement: Attachment — Submitted filename: Response to reviewers PONE-D20-28185 definitief mei 2021.docx [file pone.0252021.s001.docx]

**Author response**

**Point-by-point reply to comments from reviewers**

***Comments to the Author*** *Reviewer #1: I have reviewed with pleasure the paper by Roumen and coauthors. This large study looks at the survival of melanoma patients stratifying them in the basis of SLN. Despite the topic is not new, the procedure is still discussed and new literature data are always welcome. The sample is quite large and the paper is well written.*

We thank the reviewer for the kind remarks.

*I suggest in order to improve the strength of the message few questions:
The definition of intermediate melanoma 1-3.5 mm, despite being used in MSLT-I is quite confusing, in fact it does not include the 3.5-4 mm melanomas, that are still considered intermediates, according to the AJCC. Please discuss the reason of this decision.*
We agree with the reviewer that the selection of intermediate thickness melanomas is not in accordance with the thickness cut-off used in the AJCC/UICC TNM-staging. However, it is in accordance with the cut-offs used in the MSLT-trials, which is very important in the comparison of our results with existing literature. So, in order to enable appropriate comparison with previous studies we decided to choose the MSLT-definition of intermediate melanomas for the current study. We now added this to the manuscript.

Furthermore, for the present study we performed some additional data collection, since information on SLNB was not routinely collected by the registry in the earliest years. If we would change the cut-off values this would mean additional data collection again, which would delay the manuscript even further.

*Statistic: I would suggest to perform an AKAIKE test to value is the model is correct
How the variables selection have been performed before the inclusion in the final model?
Have the authors considered the different therapeutical approach when including the variables in the final model?*

We agree with the reviewer that we did not extensively describe the procedure that led to our final statistical model. We now added this to the manuscript.

We now described that we included variables in the model according to stepwise forward regression analyses, using the Akaike Information Criterion to choose the best fitted model. We used the stepwise options for entering variables into the model, and we chose the model with the best fit.

*Reviewer #2: The authors report a retrospective study of melanoma patients treated by SLNB or nodal observation in the period 2010-2106 with the objective to define whether SLNB could have an impact on overall survival. The adoption of SLNB for the treatment of melanoma is widely recognized in guidelines from all over the world, also considering that new adjuvant treatments can be performed only in the presence of SLNB positivity. The retrospective design of the study and the fact that the two patient groups are selected only on the basis of surgeon or patient decision to perform or not SLNB largely reduces the statistical significance of the results. Overall, the significance and clinical impact of these data in the daily practice is low as the discussion now is not whether to perform or not SLNB but rather whether to perform or not completion dissection.*
*Synopsis: in the daily clinical practice… the finding of a significant 10-year survival is not related to the daily clinical practice, rather influences daily clinical practice supporting the adoption of SLNB*
We agree with the reviewer that in current practice there is not much debate on whether or not to perform a SLNB. In the Netherlands from 2019 onwards a positive lymph node >1mm allows for adjuvant immune- or targeted therapy. However, during the period described in the current study there was much discussion on the usefulness of performing SLNB, besides it being a valuable staging tool. The natural variation that occurred from surgeons that believed in the SLNB procedure, and those that did not, allows for this type of study. The finding of survival differences prior to the introduction of adjuvant treatment, allows for placing in perspectives the survival benefits of adjuvant treatments.

(See also reply to next comment, please).


*Introduction, line 82: Due to this non distinctive primary outcome the value of
83 performing a SLNB in melanoma patients still remains a subject of great debate. Even considering the lack of 10-year survival differences between SLNB and nodal observation, SLNB biopsy represents standard of care in melanoma patients , thus its values can not be considered subject of great debate, also based on the possibility according to new data, to perform adjuvant treatments only in the presence of SLNB positive results. The authors should comment on it.*
We agree with the reviewer that in current practice there is not much debate on whether or not to perform a SLNB. However, during the study period it was. We now rephrased this accordingly in the manuscript.

*Line 84: this point is crucial but this distinction is relevant when considering completion dissection vs observation in SLNB positive patients rather than SLNB biopsy versus nodal observation in primary melanoma patients. The authors should comment on this issue.*

We agree with the reviewer that we should state more clearly that we do not study observation and lymph node dissection among SLNB-positive patients, but we compare the survival of patients who undergo a SLNB as part of their first primary melanoma diagnosis and treatment. We now described more extensively that we divided the study population into two groups based on intention-to-treat during the first 6 months after their melanoma diagnosis: patients undergoing wide local excision only (Observation group) or those who received a wide local excision combined with a SLNB (SLNB group). Patients were analyzed according to their initial grouping, irrespective of possible lymph node dissection later during the course of their disease.

*Materials and Methods: line 105: 1.2 mm – 3.5 Breslow thickness : even if this Breslow interval was that chosen in the MSLT trial, nevertheless as it does not have a specific stage definition in TNMB classification , its relevance in the daily practice is questionable considering the time lapsed from the report of MSLT 1 results.*
We agree with the reviewer that the selection of intermediate thickness melanomas is not in accordance with the thickness cut-off used in the AJCC/UICC TNM-staging. However, it is in accordance with the cut-offs used in the MSLT-trials, which is very important in the comparison of our results with existing literature. Furthermore, in clinical practice the absolute Breslow-thickness is always considered, not only the TNM-staging categories (which may differ over time across different TNM-editions). See also our reply to similar comment by reviewer #1, please.

*Line 122: The follow-up data were completed until January 1st 2018. Follow-up should be updated to 2020*.

We now extended the follow-up until February1^st^ 2021, which is the most recent date that follow-up was completed within the NCR. All analyses have been repeated based on the most recent update. This is also described in the manuscript.

*Line 137: The study population was divided into two groups: patients undergoing wide local excision only (Observation group) or those who received a wide local excision combined with a SLNB (SLNB group). This is a crucial point for the study as this statement introduces a wide range of biases and heterogeneity in the patient characteristics between these two groups that significantly reduces the statistical significance of the results. The authors should carefully discuss this issue and statistical adequate analyses to overcome this bias should be considered. Moreover, as the authors state that since 2016 SLNB biopsy even in Netherlands has become standard of care, the relevance of these results is potentially clinically limited.*

We agree with the reviewer that in current practice there is not much debate on whether or not to perform a SLNB. However, during the period described in the current study there was much discussion on the usefulness of performing SLNB. Although SLNB was considered essential for adequate staging since 2016, it was performed in only 73% of the eligible patients with a melanoma on trunk or limbs. This shows that there was still variation that probably also occurred from surgeons that believed in the SLNB procedure, and those that did not. Other factors that may have played a role, such as the age of the patient, were included in the multivariate analyses. We now described that we included variables in the model according to stepwise forward regression analyses, using the Akaike Information Criterion to choose the best fitted model. We used the stepwise options for entering variables to the model, and we chose the model with the best fit.

*RESULTS line 175: patients in the observation group were elderly and with more comorbidities , thus highlighting the differences between the two groups .*
We agree with the reviewer. This is also the reason why both age and comorbidity are included in the multivariate model. Thus, for these and other confouding factors a correction has been made by using this type of analysis. This is described more extensively in the Methods section of the manuscript.

*Line 185: the percentage of patients undergoing full lymph node dissection after a positive SLNB was apparently lower than expected (64%) , the authors should comment on this issue.*

The SLNB was not strongly recommended before 2012 for any melanoma, it was mostly considered to be a tool to obtain additional information on stage of disease. Since finding positive nodes should result in lymph node dissection, which may lead to complications or morbidity, without real proof of prolonged survival, some doctors preferred not to perform the procedure.

During the study period, there was no clear evidence that full lymph node dissection after a positive SLNB resulted in better survival rates. However, the risk of morbidity after a full lymph node dissection was well described, so patient- and/or doctor-preferences may have led to not performing the lymph node dissection after a positive SLNB. This is now described in the manuscript. (See also comment on paragraph above starting with Line 84, please).

*Line 188 Survival , the authors considered overall survival which could be a controversial finding considering that the group of patients undergoing only nodal observation is characterized by an elderly age. Could the authors provide data on disease-specific survival ?*

Melanoma specific survival would perhaps be a better way to reflect the therapeutic effect of SLNB, however, unfortunately the Netherlands Cancer Registry does not have access to cause of death. As a measure of disease-specific survival we calculated relative survival (Dickman* et al, 2004), data not shown in the manuscript, see figure below. In relative survival the ratio of the mortality in the studied population for each year, age and gender to the mortality in the background population is calculated.

This also shows a clear distinction between those patients who underwent SLNB and those without SLNB. The estimate for risk for death (Relative Excess Risk, RER) was significantly lower for patients who underwent SLNB. Since these results are rather similar to those of the crude survival, we chose to present the latter, which we considered to be easier to interpret.

Furthermore, the more easily interpretable hazard ratios, that are presented in Table 2 in the manuscript, show the hazards for each variable adjusted for all others in the table, also including gender, age and comorbidity.

If the reviewer or editor decides that relative survival would be a better way to present our results, we would be happy to replace Figure 2 by the figure below, or even add it to the paper.

| **Parameter** | **Level** | **Estimated RER** | **Lower limit** | **Upper limit** |
| --- | --- | --- | --- | --- |
|  |  |  | **95% CI** | **95% CI** |
| **SWK** | SNLB | 0,69 | 0,54 | 0,88 |
|  | No SLNB | ref |  |  |

*Dickman, P. W., Sloggett, A., Hills, M. and Hakulinen, T. (2004), Regression models for relative survival. Statist. Med., 23: 51–64.

*Line 202: post hoc external validation: the control group of the Netherlands Cancer Registry should be described in terms of clinico-pathologic characteristics and compared to identify potential differences with respect to that of the Eindhoven region. Considering overall survival rates, the survival of SLNB patients in the Eindhoven group (75%) was similar to that of the observation group in the Netherlands Cancer Registry (72%) thus implying that the impact of SLNB procedure is definitely debatable based on these data.*

We agree with the reviewer that when comparing survival rates, the other factors important for survival should also be compared. Unfortunately, comorbidity was not available at a nation-wide level. However, age and gender were distributed comparably. This is now also described in the manuscript.

*It should be considered the survival of SLNB positive patients and compared with that of patients diagnosed with nodal metastases after observation.*

We agree that it would be interesting to compare survival of SLNB positive patients with that of patients with nodal metastases after observation. Within the cancer registry, information related to the primary diagnosis and treatment is available, but information on disease progression is not routinely collected. Information on vital status is updated each year, so we can only provide survival rates related to patient- and tumor characteristics, as well as treatment modalities.

Finally, as some time has passed since our first manuscript, we updated the information on literature on this subject, that is displayed in table 3. This is also mentioned in the discussion section.
